# The reason why thin-film silicon grows layer by layer in plasma-enhanced chemical vapor deposition

**DOI:** 10.1038/srep09052

**Published:** 2015-03-16

**Authors:** Takuya Kuwahara, Hiroshi Ito, Kentaro Kawaguchi, Yuji Higuchi, Nobuki Ozawa, Momoji Kubo

**Affiliations:** 1 Fracture and Reliability Research Institute (FRRI), Graduate School of Engineering, Tohoku University, 6-6-11 Aoba, Aramaki, Aoba-ku, Sendai, Miyagi 980-8579, Japan

## Abstract

Thin-film Si grows layer by layer on Si(001)-(2 × 1):H in plasma-enhanced chemical vapor deposition. Here we investigate the reason why this occurs by using quantum chemical molecular dynamics and density functional theory calculations. We propose a dangling bond (DB) diffusion model as an alternative to the SiH_3_ diffusion model, which is in conflict with first-principles calculation results and does not match the experimental evidence. In our model, DBs diffuse rapidly along an upper layer consisting of Si-H_3_ sites, and then migrate from the upper layer to a lower layer consisting of Si-H sites. The subsequently incident SiH_3_ radical is then adsorbed onto the DB in the lower layer, producing two-dimensional growth. We find that DB diffusion appears analogous to H diffusion and can explain the reason why the layer-by-layer growth occurs.

Thin-film Si has attracted attention as a promising material for solar cells^1^. Plasma-enhanced chemical vapor deposition (PECVD) using SiH_4_ is a key technique for fast large-area growth at low temperatures^2^,^3^,^4^,^5^. Tsai *et al.* showed that SiH_3_ radicals are the predominant deposition precursors and effective for forming “device-quality” films, that is, films with atomically smooth surfaces^2^,^3^. Obtaining smooth surfaces is particularly important for practical applications because rough surface morphologies induce surface and interface defects and reduce carrier lifetimes. Elucidation of the mechanisms by which layer-by-layer growth of thin-film Si occurs in PECVD is therefore strongly desired for the development of high-performance solar cells.

Experimental studies of surface morphologies have suggested reasons behind the surface smoothing of thin-film Si^3^,^6^,^7^,^8^,^9^,^10^,^11^. It is well known that surface diffusion of deposition precursors is essential for explaining the surface smoothing mechanism in simple homoepitaxial growth using molecular beam epitaxy (MBE)^12^. Kukushkin *et al*. performed remarkable and important works on layer-by-layer growth mechanisms of thin-film in epitaxial growth^13^,^14^,^15^,^16^. They proposed the theory of layer-by-layer growth by diffusional coalescence of faceted islands both on a crystal surface and on an island containing screw dislocations. Then, they theoretically demonstrated that the tablet- and needle-like growth of thin-film take place when the main mode of mass-transport is two- and three-dimensional diffusion on the substrate, respectively. Furthermore, there is almost no doubt that surface diffusion produces smooth surfaces in CVD because experimental results have revealed a strong dependence of surface roughness on substrate temperatures and growth rates^3^,^17^. Matsuda *et al.* found a correlation between surface loss probabilities of SiH_3_ radicals and surface H coverage and suggested a three-center diffusion model^11^,^17^. In that model, SiH_3_ radicals are physisorbed onto Si-H sites and diffuse along the hydrogenated surface. Although the three-center diffusion model has been accepted to some extent, scaling behavior analyses of surface morphologies using atomic force microscopy (AFM) and *in situ* ellipsometry have not found physisorption sites for SiH_3_ radicals^18^. Additionally, diffusion models remain an indirect explanation of the smoothing mechanism because experimental observations of surface reactions at the atomic scale are difficult. Computational simulations are thus effective for gaining a direct understanding of the surface reactions in PECVD. Maroudas *et al.* suggested a “valley-filling” mechanism based on molecular dynamics (MD) simulations^19^. They referred to microscopic higher- and lower-deposition areas as surface hills and valleys, respectively, and suggested that SiH_3_ radicals diffuse from surface hills to valleys and preferentially passivate dangling bonds (DBs) located in surface valleys. They argued that SiH_3_ radicals are weakly adsorbed on the hydrogenated surface. However, Cereda *et al.* used density functional theory (DFT) calculations to show that SiH_3_ radicals abstract H atoms by overcoming the negligible small activation energy^20^. This means that SiH_3_ radicals cannot exist on a hydrogenated surface in the weakly physisorbed state. Long-distance diffusion of weakly physisorbed SiH_3_ radicals is therefore unlikely. We also found from tight-binding quantum chemical MD simulations that the initial growth of thin-film Si follows an “abstraction-adsorption” mechanism^21^,^22^, where a DB is generated on the surface via H abstraction by a SiH_3_ radical and the next SiH_3_ radical is adsorbed onto the generated DB. Thus, although experimental results have found a relationship between surface diffusion and layer-by-layer growth^23^,^24^, the diffusion mechanism of SiH_3_ radicals has not yet been confirmed, and the previously proposed diffusion mechanisms thus remain controversial. In particular, the questions of what species diffuses and how the surface diffusion affects the formation of atomically smooth surfaces are still open to debate. Against this background, we perform self-consistent-charge density-functional tight-binding (SCC-DFTB)^25^,^26^ MD and DFT calculations and report a surface diffusion mechanism that can explain how smooth surfaces are formed by Si PECVD. An understanding of the layer-by-layer growth mechanism is expected to enable atomic-scale design of PECVD processes for producing thin-film Si with atomically smooth surfaces leading to low defect densities and high photostability.

SCC-DFTB MD simulations of a Si(001)-(2 × 1):H surface were performed by continuous impingement of 25 SiH_3_ radicals at intervals of 2.0 ps. We calculated 15 trajectories for durations of up to 50 ps. Figure 1 shows snapshots of a CVD simulation of a typical trajectory. On the Si(001)-(2 × 1):H surface, an incident SiH_3_ radical abstracts a H atom and generates a SiH_4_ molecule (Fig. 1(a) and (b)). A DB is also generated on the surface. The subsequently incident SiH_3_ radical is then adsorbed onto the DB (Fig. 1(c) and (d)). This is in agreement with our previously proposed “abstraction-adsorption” mechanism^22^. At this point, we define the terms lower layer and upper layer for referring to the layers of Si atoms terminated by H atoms. The lower layer is defined as the top layer of Si atoms of the original Si(001)-(2 × 1):H surface, and the upper layer is defined as the layer above the lower layer and consists of SiH_3_ sites generated by the abstraction-adsorption process. After the first 24.0 ps of simulation time, four SiH_3_ radicals have been adsorbed onto the surface via the abstraction-adsorption mechanism. Next, an incident SiH_3_ radical abstracts a H atom from an adsorbed SiH_3_ species (Fig. 1(e) and (f)). The reaction generates a Si-H_2_ site with a DB in the upper layer (Fig. 1(g)). The Si-H_2_ site abstracts a H atom from a neighboring Si-H site in the lower layer. This effectively means that the DB migrates from the upper layer to the lower layer (Fig. 1(h)). The Si-H_3_ site thus re-forms via the DB migration, with a DB newly generated in the lower layer. Over the course of 15 MD trajectories, DB migration from the upper layer to the lower layer is observed six times, while migration from the lower layer to the upper layer is observed only once. Another incident SiH_3_ radical is then adsorbed onto the DB located in the lower layer (Fig. 1(i) and (j)). The next incident SiH_3_ radical abstracts a H atom from an adsorbed SiH_3_ species, again generating a Si-H_2_ site with a DB in the upper layer (Fig. 1(k)). The Si-H_2_ site abstracts a H atom from a neighboring Si-H_3_ site. The DB migrates from the Si-H_2_ site to a neighboring Si-H_3_ site in the same layer (Fig. 1(l)). We find that DB migration can be classified into three patterns: (1) from a Si-H_2_ site in the upper layer to a Si-H site in the lower layer; (2) from a Si site in the lower layer to a Si-H_3_ site in the upper layer; and (3) from a Si-H_2_ in the upper layer to a Si-H_3_ site in the same layer. These patterns of DB migration occur in 23%, 4%, and 73% of the 15 MD trajectories, respectively. However, we do not observe physisorption or surface diffusion of SiH_3_ radicals, though these phenomena were previously suggested by experimental groups^11^. Our simulation shows that physisorbed SiH_3_ radicals do not migrate whereas DBs frequently do so on the hydrogenated surface.

To validate the migration of DBs from a Si-H_2_ site in the upper layer to a neighboring Si-H site in the lower layer on Si(001)-(2 × 1), which corresponds to pattern (1) described above, we calculated the activation energies of three possible migration paths from a step site using DFT. In Fig. 2(a), P1, P2, and P3 represent the migration of a DB between two adjacent dimer rows, between two adjacent dimers in the same dimer row, and within the dimer, respectively. Figure 2(b) shows that the activation energies of P1, P2, and P3 are 8.29, 11.66, and 19.27 kcal/mol, respectively. However, the reverse reactions of P1, P2, and P3 require higher activation energies of 15.65, 19.02, and 26.63 kcal/mol, respectively, which correspond to pattern (2). This means that DBs stay in the lower layer for long periods of time. Moreover, our DFT calculations show that it is difficult for DBs to diffuse along the lower layer, which consists of Si-H sites, due to the high activation energy of 43.85 kcal/mol, and such diffusion is not observed in our SCC-DFTB MD simulations. The DBs thus remain at step sites in the lower layer. This indicates that the next SiH_3_ radical could be adsorbed onto a step site in the lower layer.

Next, we calculated the activation energies for the migration of DBs from a Si-H_2_ in the upper layer to a Si-H_3_ site in the same layer via three possible migration paths corresponding to pattern (3). In Fig. 3(a), P4, P5, and P6 represent the migration of a DB between two adjacent dimer rows, between two adjacent dimers in the same dimer row, and within the dimer, respectively. Our DFT calculations yield activation energies of 1.37, 4.47, and 9.08 kcal/mol for P4, P5, and P6, respectively. These values are much smaller than those for migration from the upper layer to the lower layer (see Fig. 2). This means that DBs migrate more rapidly within the upper layer than from the upper layer to the lower layer. This is in good agreement with our SCC-DFTB MD calculations.

Our calculations find that, in addition to the adsorption of SiH_3_ radicals via an abstraction-adsorption mechanism^22^, the DBs on the hydrogenated surface exhibit three important behaviors: (a) DBs migrate from a Si-H_2_ in the upper layer to a Si-H_3_ site in the same layer; (b) DBs migrate from a Si-H_2_ site in the upper layer to a Si-H site in the lower layer; and (c) DBs remain on step sites in the lower layer for a long duration. Figure 4 shows a model for the layer-by-layer growth mechanism based on the above results. First, the abstraction-adsorption mechanism occurs repeatedly, resulting in SiH_3_ radicals gradually adsorbing onto the surface (Fig. 4(a) and (b)). Terakawa *et al.* suggested that the topmost layer is covered with SiH_3_ species^27^. We also suggest the formation of an “island” structure consisting of adsorbed SiH_3_ radicals (Fig. 4(c)). The surface thus becomes divided into terraces of lower layers and upper layers, which consist of Si-H and Si-H_3_ sites, respectively. Second, a H atom is abstracted from a Si-H_3_ site, generating a Si-H_2_ site with a DB on the upper terrace (Fig. 4(c)). Third, the DB rapidly diffuses along the upper terrace until it finally reaches a step edge (Fig. 4(d)). Fourth, the DB migrates from the upper layer to the lower layer at the step edge (Fig. 4(e)). The residence time of the DB at the step site in the lower layer is much longer than in the upper layer because the configuration in Fig. 4(e) is more stable than that in Fig. 4(d). A DB at a step site in the lower layer does not diffuse to the lower terrace, but instead stays there due to the high energy barrier. The next incident SiH_3_ radical can thus be adsorbed onto the DB at the step site in the lower layer, resulting in lateral growth (Fig. 4(f)). If the DB does not migrate but instead stays in the upper layer, the next incident SiH_3_ radical is adsorbed on the upper layer, and forms a longitudinal Si-Si bond. The occurrence of such longitudinal growth before sufficient lateral growth increases the surface roughness. According to our model, DB migration prevents the adsorption of incident SiH_3_ radicals onto the upper layer and produces growth of the lower layer. This thus shows that DB migration from the upper layer to the lower layer is necessary for layer-by-layer growth. During DB diffusion, H atoms also diffuse in the opposite direction to the DBs. Interestingly, the DB diffusion appears analogous to H diffusion. Although it was previously thought that layer-by-layer growth occurs due to diffusion of the SiH_3_ radical deposition precursors^11^,^17^, we newly propose that DBs are the diffusing species and the DB diffusion produces the layer-by-layer growth.

Experimental results have shown that the surface diffusion of DBs during the interval between impingements of SiH_3_ radicals on the upper layer strongly influences surface roughness^3^,^17^. To reduce the surface roughness, we thus need to investigate how long the DBs can diffuse on the upper layer for a given terrace size during the time interval between impingement of SiH_3_ onto the upper layer. To estimate the diffusion length, we calculated the time needed for DB diffusion from the upper layer to the lower layer depending on the terrace size of the upper layer. To do this, we numerically analyzed the surface diffusion of a DB from the upper layer to the lower layer using transition-state theory. In our numerical calculations, we follow the time evolution of the existence probability of a DB and estimate the time needed for the DB to diffuse from the upper layer to the lower layer. We consider only DB diffusion in the calculation; impingement of SiH_3_ radicals is not considered. The time interval between impingements of SiH_3_ radicals onto the upper layer was estimated from experimental data for SiH_3_ radical densities in plasma. Figure 5(a) shows the calculation model, which consists of a SiH_3_ island on a Si(001) surface with dimers. In Fig. 5(a), orange circles represent H-terminated Si (Si-H) sites consisting of top-layer Si atoms on a Si(001) surface and green circles represent Si-H_3_ sites consisting of SiH_3_ species adsorbed on the top-layer Si atoms. These correspond to the lower and upper layers, respectively. DB diffusion from a step site in the lower layer to the terrace in the lower layer is negligible due to the high energy barrier of 43.85 kcal/mol, which corresponds to a residence time of approximately 400 h at 500 K. We therefore consider only step sites in the lower layer in the model as shown in Fig. 5(a). The jump rate 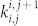 from the *i*th row and *j*th column site *P_i,j_* to an adjacent site *P_i,j+1_* is calculated using the Arrhenius equation,
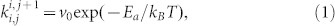
where *k_B_* is the Boltzmann constant, *T* is the substrate temperature, *E_a_* is the activation energy, and *v_0_* is a pre-exponential factor. The standard value^28^ *v_0_* = 1.0 × 10^13^ s^−1^ and substrate temperatures *T* = 300–900 K are used. We assume that the temperatures of adsorbed SiH_3_ radicals are equal to the substrate temperature *T*. We use the activation energies obtained by our DFT calculations in Figs. 2 and 3. The activation energies depend on the diffusion directions, and the activation energies of DB diffusion from the upper to lower layers are different from those of DB diffusion from the lower to upper layers. According to transition-state theory, the time derivative of the existence probability *θ_i,j_* (0 ≤ *θ_i,j_* ≤ 1) of a DB at site *P_i,j_* is given by the sum of the inflows from adjacent sites and outflows to adjacent sites:
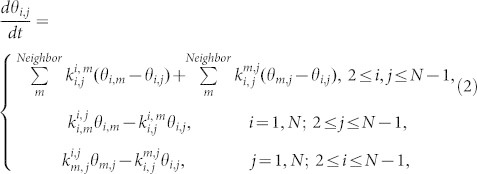
Here, *m* is an index that related to an adjacent site, and *N* is the length of each side in the calculation model. For 2 ≤ *i, j* ≤ *N* − 1, the site belongs to the upper layer; otherwise, the site belongs to the lower layer. The top equation corresponds to the derivatives for sites in the upper layer, and the second and third equations correspond to the derivatives for sites in the lower layer. These are integrated numerically using a fourth-order Runge-Kutta method for a maximum of 0.1 s. We consider only the case of an odd number *N*. Since the generation of DBs can be expected to be random in the upper layer, we assume the existence probabilities of DBs at all sites in the upper layer are equal at *t* = 0.0 s; that is, 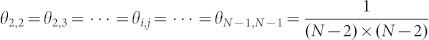 (2 ≤ *i, j* ≤ *N* − 1). At *t* = 0.0 s, the existence probabilities at all sites in the lower layer are equal to 0. The total probability for all sites including both the lower and upper layers is equal to 1 at *t* = 0.0 s, and this is conserved during the numerical calculations. The calculations are terminated when the total probability for all sites in the lower layer reaches 0.9. We define the time until the calculation terminates as the time needed for DB diffusion from the upper layer to the lower layer. Figure 5(b) shows the time needed for the total probability of the lower layer to reach 0.9 as a function of substrate temperature and *N*. The dependency on *N* is important because SiH_3_ island size increases with time during actual CVD growth. First, we focus on the behavior of the existence probability at low temperatures (less than 700 K). The time needed for the total probability of the lower layer to reach 0.9 increases as the size of the SiH_3_ island increases and as the substrate temperature decreases. To check the validity of our calculations, we compare the diffusion length obtained from our numerical calculation results with that obtained from experimental results. The diffusion length strongly depends on the time interval between incident SiH_3_ radicals reaching the upper layer because DBs have to diffuse from the upper layer to the lower layer before the next SiH_3_ radical impinges on the upper layer in order for lateral growth to occur. We estimate the diffusion length using both the necessary time calculated above and the time interval obtained from experimental data on SiH_3_ radical densities in plasma. Experimentally, the cavity ring down technique for detection of SiH_3_ radicals found high SiH_3_ radical densities^29^ of 2.0 × 10^18^ to 1.2 × 10^19^ m^−3^. From these results, we estimate that SiH_3_ radicals reach the surface every 1.8 × 10^−6^ s per area of 10.0 × 10.0 nm^2^ at a SiH_3_ radical density of 1.0 × 10^19^ m^−3^ at 400 K. The area of 10.0 × 10.0 nm^2^ is almost equal to the SiH_3_ island size of *N* = 29 considering that there is one Si-H_3_ site per area of 0.384 × 0.384 nm^2^ on Si(001) surface. Figure 5(b) shows that the necessary time is about 1.8 × 10^−6^ s at 400 K when *N* = 29, which corresponds approximately to the time interval of 1.8 × 10^−6^ s per area of 10.0 × 10.0 nm^2^. Therefore, for *N* ≤ 29, a DB can diffuse from the upper layer to the lower layer before the next SiH_3_ radical reaches the SiH_3_-island at 400 K, thus allowing further lateral growth. When *N* = 29, at which point the length of each side of the SiH_3_ island is about 10 nm, DBs diffuse between 2.5 and 5.0 nm along the dimer row direction on average. At 500 K, when *N* = 51, the island size is almost equal to 20.0 × 20.0 nm^2^, and the necessary time corresponds approximately to a time interval of 4.4 × 10^−7^ s per area of 20.0 × 20.0 nm^2^. The average diffusion length thus increases to 5.0–10 nm at 500 K. Collins *et al.* showed that the diffusion length is between 6 and 10 nm at about 500 K by real-time spectroscopic ellipsometry^23^, which is in good agreement with our results. Next, we focus on the behavior of existence probabilities at high temperatures (greater than 800 K). In Fig. 5(b), the lines at 800 and 900 K stop at *N* = 43 and 27, respectively, which indicates that the existence probabilities did not reach 0.9 within 0.1 s. The behavior of the existence probabilities at greater than 800 K is significantly different from that at less than 700 K. In Fig. 5(b), the time needed for the total probability of the lower layer to reach 0.9 at greater than 800 K is separated into four regions by inflection points and discontinuity with respect to *N*. In Region I, DBs diffuse rapidly from the upper layer to *P*_1,*j*_, *P_N_*_,*j*_, and *P_i_*_,1_ (2 ≤ *i, j* ≤ *N* − 1) in the lower layer. The slopes are the same as those at less than 700 K. In Region II, the DBs generated at *P*_1,*j*_, *P_N_*_,*j*_, and *P_i_*_,1_ (2 ≤ *i, j* ≤ *N* − 1) in the lower layer return to the upper layer because the inverse reactions of P1 and P2 are likely to occur before the DBs diffuse to the lower layer in a large SiH_3_ island at high temperatures. The necessary time thus increases drastically. In Region III, DBs diffuse from the upper layer to *P_i,N_* (2 ≤ *i* ≤ *N* − 1) in the lower layer because the reaction of P3 is likely to occur at high temperatures. This again reduces the slopes. In Region IV, although the reactions are the same as in Region III, the existence probabilities in the upper layer increase because it is difficult for the DBs to reach the step edges in the large SiH_3_ island, and the DBs thus remain on the upper layer. Therefore, at 900 K, the existence probability in the lower layer is unable to reach 0.9 when *N* = 29. As a consequence, an interesting phenomenon occurs where the necessary time increases discontinuously between *N* = 27 and 29. Our results indicate that the increase in substrate temperatures causes the return of DBs to the upper layer, which decreases the existence probability of DBs in the lower layer, because DBs stay on the upper layer for a long period of time when the SiH_3_ island size is large. As Figure 5(b) shows, the DBs start to return to the upper layer at 800 K, a phenomenon that becomes more pronounced as the substrate temperature increases. This results in the adsorption of SiH_3_ radicals onto the upper layer and an increase in surface roughness. Kondo *et al.* used AFM to show that surface roughness increases with increasing substrate temperatures^2^, which is consistent with our calculation results. Taken together, our results show how DBs diffuse a long distance and what substrate temperatures are suitable for obtaining atomically smooth surfaces.

In PECVD processing of thin-film Si, it was previously believed that the SiH_3_ radical deposition precursors were the diffusing species. This was based on the fact that the precursors of Si adatoms diffuse on the surface during MBE deposition^12^. However, this is inconsistent with DFT calculation results^20^ and has no supporting experimental evidence. In this article, DBs are found to be the diffusing species in PECVD. Flewitt *et al.* studied the evolution of the surface topography using *in situ* scanning tunneling microscopy^9^ and proposed that preferential creation of DBs at step sites produces smooth surfaces, which is consistent with our results. However, they could not explain the long-distance diffusion of DBs, that is, how DBs on the terraces arrive at step edges. In contrast, the DB diffusion mechanism proposed in this article is able to explain the overall process of layer-by-layer growth.

In summary, we have used SCC-DFTB MD and DFT calculations to elucidate the reason why thin-film Si grows layer by layer in PECVD. DBs diffuse rapidly along the upper layer, which is covered with Si-H_3_ sites. When the DBs reach step edges, they move down an atomic step. Owing to the longer residence time of the DBs at step sites in the lower layer, the next SiH_3_ radical is adsorbed onto a step site in the lower layer, leading to lateral growth. We propose that this DB diffusion mechanism explains the layer-by-layer growth in Si PECVD.

## Methods

We employ the SCC-DFTB MD method to simulate the CVD growth dynamics of thin-film Si at a finite temperature. The detailed methodology of SCC-DFTB is described elsewhere^25^,^26^. The Si(001)-(2 × 1) surface has a slab geometry, and consists of 6 Si layers with 16 atoms per layer. The top and bottom layers are terminated with 32 H atoms. The slab model has a vacuum region of 22.7 Å. The temperature in SCC-DFTB MD simulations is set at 500 K. A time step of 0.1 fs is used. Fractional orbital occupations following the Fermi-Dirac distribution are employed with an electronic temperature of 1500 K^30^. DFT calculations are performed within the generalized gradient approximation and Perdew-Wang exchange-correlation functional using the DMol^3^ code^31^. Spin-polarization is taken into account. Double numerical plus polarization is used as basis set with a global space cutoff of 4.6 Å. Three-dimensional periodic boundary conditions using the Gamma-point approximation and an effective core potential method are applied^32^. A transition state search scheme is employed based on a combination of traditional LST/QST methods^33^.

## Author Contributions

T.K. and M.K. planed and supervised the study, and developed the original concept. T.K. developed the simulator and performed the simulations and data analysis. T.K. and Y.H. designed the theoretical model. T.K., H.I., K.K., Y.H., N.O. and M.K. discussed the results. T.K. wrote the manuscript, and Y.H. and M.K. revised the manuscript.

## Figures and Tables

**Figure 1:**
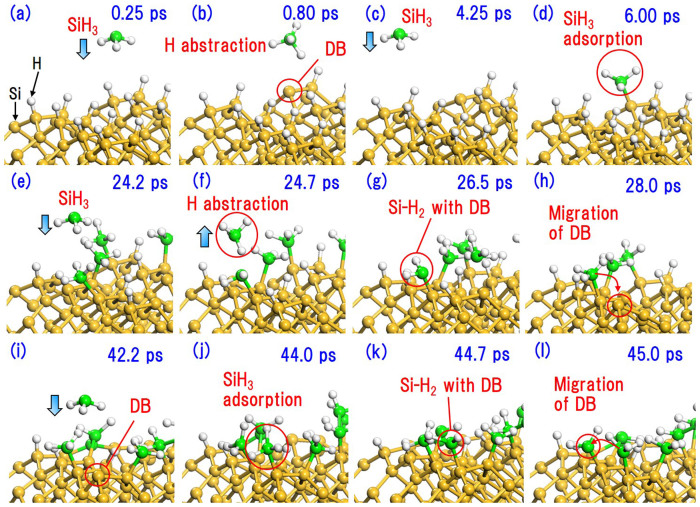
Snapshots of a Si CVD simulation. (a) Before and (b) after H abstraction from a Si-H site by a SiH_3_ radical. (c) Before and (d) after adsorption of a SiH_3_ radical onto a DB. (e) Before and (f) after H abstraction from a Si-H_3_ site by a SiH_3_ radical. (g) Generation of a Si-H_2_ site with a DB. (h) DB migration from the upper layer to the lower layer. (i) Before and (j) after adsorption of a SiH_3_ radical onto the lower layer. (k) Before and (l) after DB migration within the upper layer.

**Figure 2:**
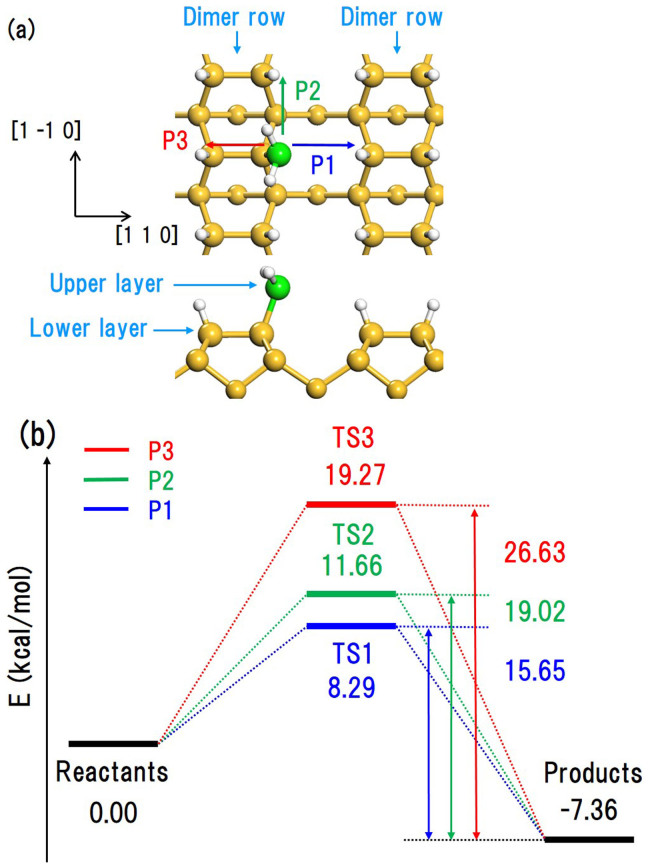
(a) Top and side views of the Si(001)-(2 × 1):H surface with an adsorbed SiH_2_. Arrows indicate migration paths of DBs from a Si-H_2_ site in the upper layer to an adjacent Si-H site in the lower layer. (b) Energy profiles of DB migration along paths P1, P2, and P3.

**Figure 3:**
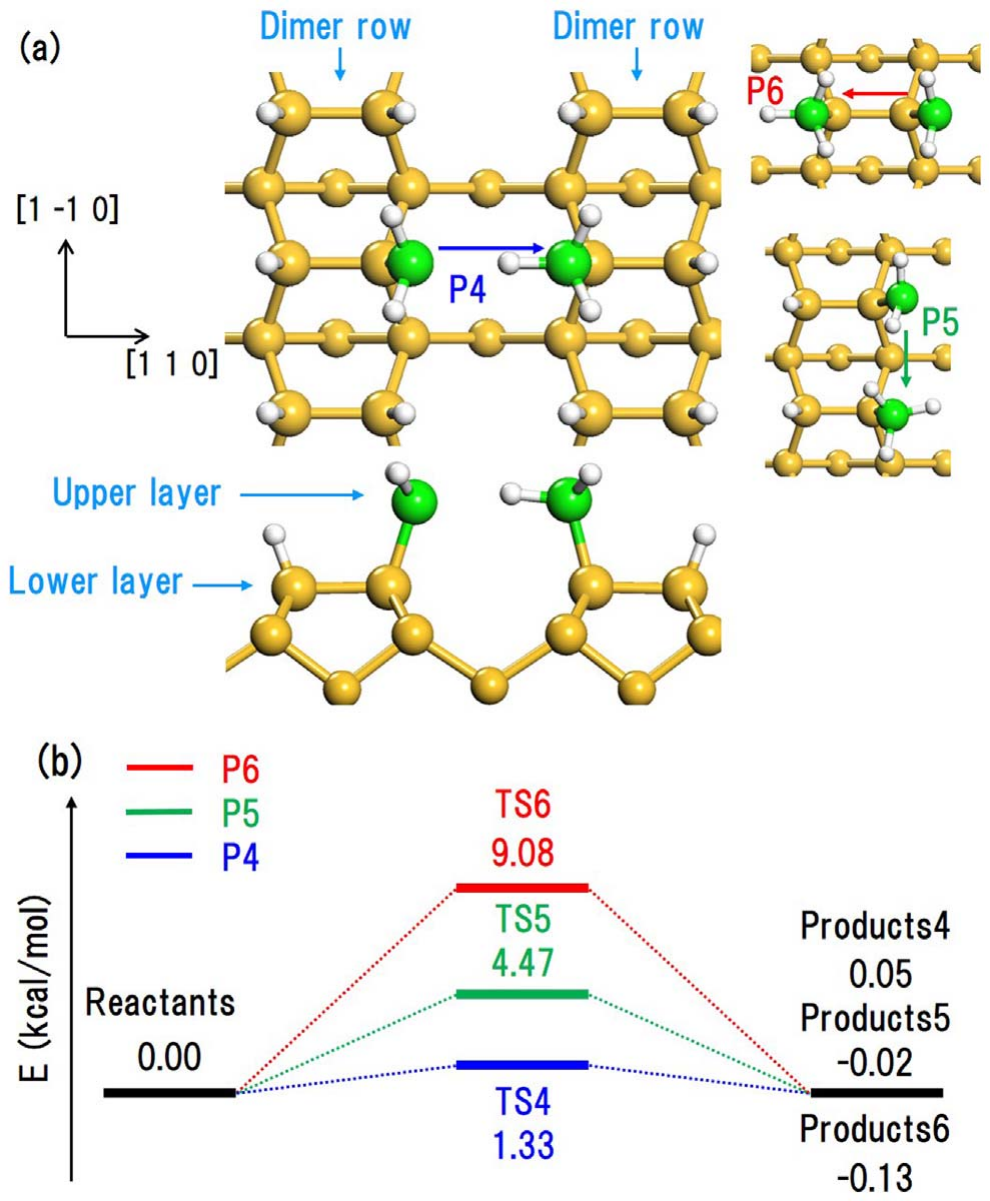
(a) Top and side views of the Si(001)-(2 × 1):H surface with an adsorbed SiH_2_ and SiH_3_. Arrows indicate migration paths of DBs from a Si-H_2_ site to an adjacent Si-H_3_ site. (b) Energy profiles of DB migration along paths P4, P5, and P6.

**Figure 4:**
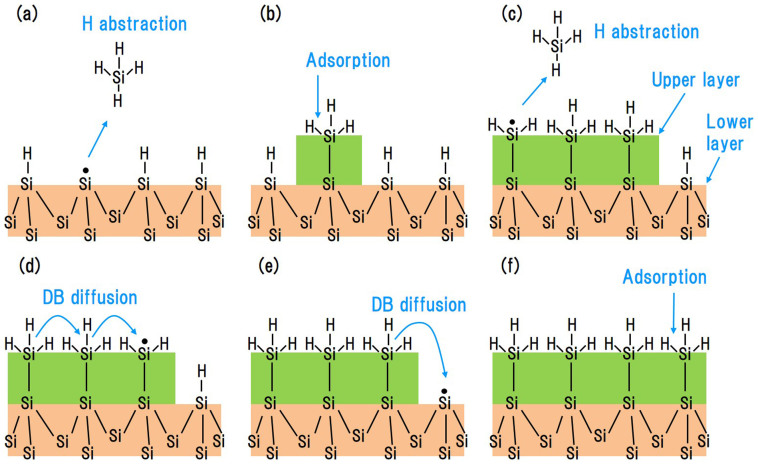
Schematic diagram of layer-by-layer growth of thin-film Si by PECVD. (a) Generation of a DB via H abstraction of a SiH_3_ radical. (b) Adsorption of a SiH_3_ radical on a DB. (c) DB diffusion within the upper layer consisting of SiH_3_ sites. (d) DB diffusion from the upper layer to the lower layer. (e) Generation of a DB in the lower layer. (f) Two-dimensional growth by the adsorption of SiH_3_ radicals onto the lower layer.

**Figure 5:**
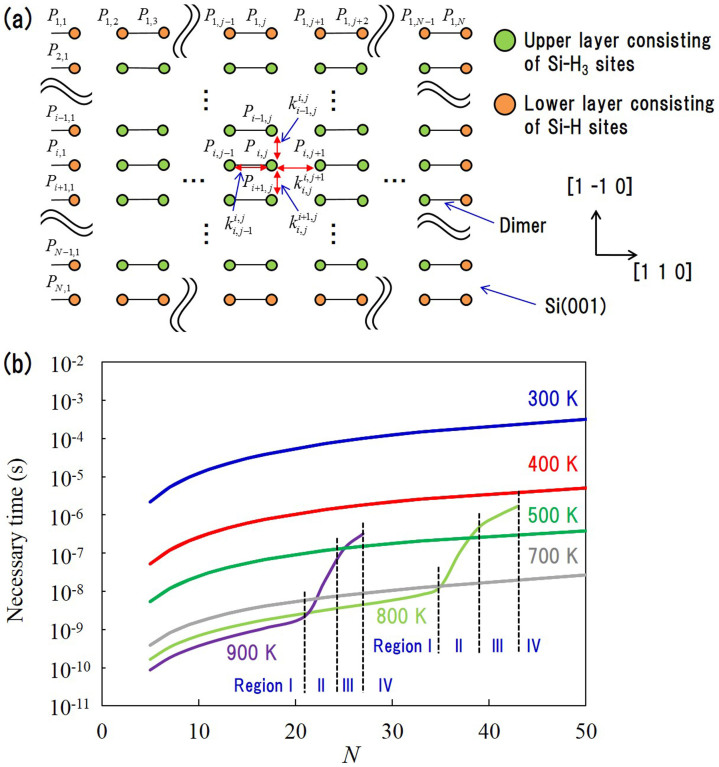
(a) Kinetic model of surface diffusion in the island consisting of Si-H_3_ sites on Si(001). (b) Time needed for DB diffusion from the upper layer to the lower layer.
